# Plant Biomass Depolymerization by Oxidoreductases: Structural Characteristics, Synergy Mechanism, and Evolutionary Adaptation

**DOI:** 10.1021/acs.jafc.5c16214

**Published:** 2026-06-25

**Authors:** Xiaoyu Ma, Florian Csarman, Yunjia Guan, Lushan Wang, Roland Ludwig, Su Ma

**Affiliations:** † State Key Laboratory of Microbial Technology, 12589 Shandong University, Binhai Road 72/N2, 266237 Qingdao, China; ‡ Institute of Food Technology, Department of Biotechnology and Food Science, BOKU University, Muthgasse 11, 1190 Vienna, Austria

## Abstract

Plant biomass, predominantly composed of lignocellulose, exhibits structural and chemical complexity that limits its biological deconstruction. This review evaluates the oxidative strategies utilized by microorganisms, primarily filamentous fungi, to overcome the recalcitrance. We integrate recent experimental breakthroughs with structural bioinformatics to map the expanding landscape of redox enzymes, including newly discovered AA families. Central enzymatic systems involved in lignin depolymerization include laccases and lignin-active peroxidases, whereas polysaccharide-degrading oxidoreductases comprise lytic polysaccharide monooxygenases (LPMOs) and their auxiliary redox partners. Hydrogen peroxide acts as a central intermediate that bridges enzymatic sources with sinks, subject to spatiotemporal regulation. The review also incorporates the synergistic interplay between oxidoreductases and nonenzymatic radical systems, offering refined mechanistic insights. These understandings underpin the design of next-generation biorefineries, accelerating the transition toward a sustainable circular bioeconomy.

## Graphical abstract

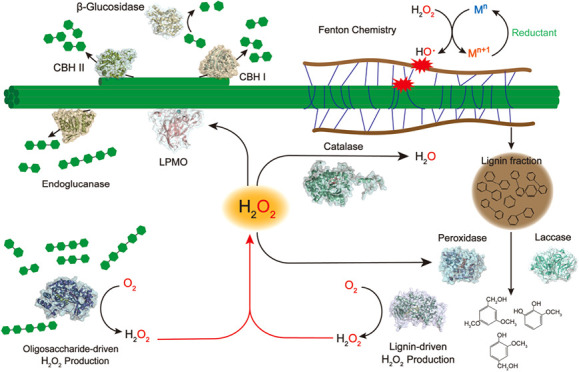

## 1 Advantages and Challenges of Enzymatic Biomass Depolymerization

The total plant biomass on Earth is approximately 470 gigatons of carbon (GtC), accounting for ∼80% of the overall biomass, with land plants (embryophytes) representing the dominant fraction.^1^ This massive carbon reservoir is sustained by an annual gross primary production (GPP) of approximately 132.6 GtC, representing the total carbon fixed through terrestrial photosynthesis.^2^ For perspective, this annual photosynthetic carbon flux is about 12.5 times larger than the combined annual global consumption of crude oil (4.2 GtC/a) and coal (6.4 GtC/a).^3^ While a significant portion of this GPP must be retained within ecosystems to sustain carbon stocks, nutrient cycles, and biodiversity, plant biomass nonetheless represents a highly renewable bioresource that can be used to produce biofuels, chemicals, and advanced materials to replace fossil-based products, thereby reducing net CO_2_ emissions by leveraging a closed carbon loop.^4^ Lignocellulose as the primary constituent of plant cell walls comprises over 50% of the dry weight of plant biomass. It is a complex, three-dimensional network based on predominately lignin, cellulose and hemicellulose (Figure 1). Lignin is the most abundant renewable source of aromatic polymers on Earth. With its high carbon content and complex aromatic structures, lignin presents immense potential for broad-ranging applications.^5^ Lignin is synthesized via the oxidative polymerization of three hydroxycinnamyl alcohol precursors, known as monolignols: *p*-coumaryl, coniferyl, and sinapyl alcohols. These precursors give rise to the *p*-hydroxyphenyl (H), guaiacyl (G), and syringyl (S) structural units, respectively, within the lignin macromolecule.^6,7^ The relative abundance of these three units varies significantly among plant lineages, with softwood (gymnosperm) lignins being predominantly composed of G-units, hardwood (angiosperm) lignins containing both G- and S-units, and grass (monocot) lignins incorporating all three (G, S, and H) units.^8^ The complex aromatic polymer is obtained through radical coupling and provides structural rigidity and protection against degradation.^8^ In cellulose, multiple linear polysaccharide chains are aligned into microfibrils via hydrogen bonds, a crystalline network that provides plants with mechanical rigidity.^9^ Hemicelluloses comprise a heterogeneous group of branched polysaccharides, such as xylans and mannans, that function as structural cross-linkers within the cell wall. These polysaccharides comprise diverse pentose and hexose residues, primarily β-d-xylosyl, β-d-mannosyl, and β-d-glucosyl units, which are frequently decorated with arabinosyl or galactosyl side chains. Within the cell wall matrix, hemicelluloses facilitate the integration of lignin and cellulose through covalent ester linkages and hydrogen bonding, respectively.^10^ Plant cell walls serve as natural barriers, exhibiting exceptional structural resilience and intrinsic resistance to biological degradation in an evolutionary arms race, which poses substantial challenges to the efficient utilization of the plant biomass.

**Figure 1:**
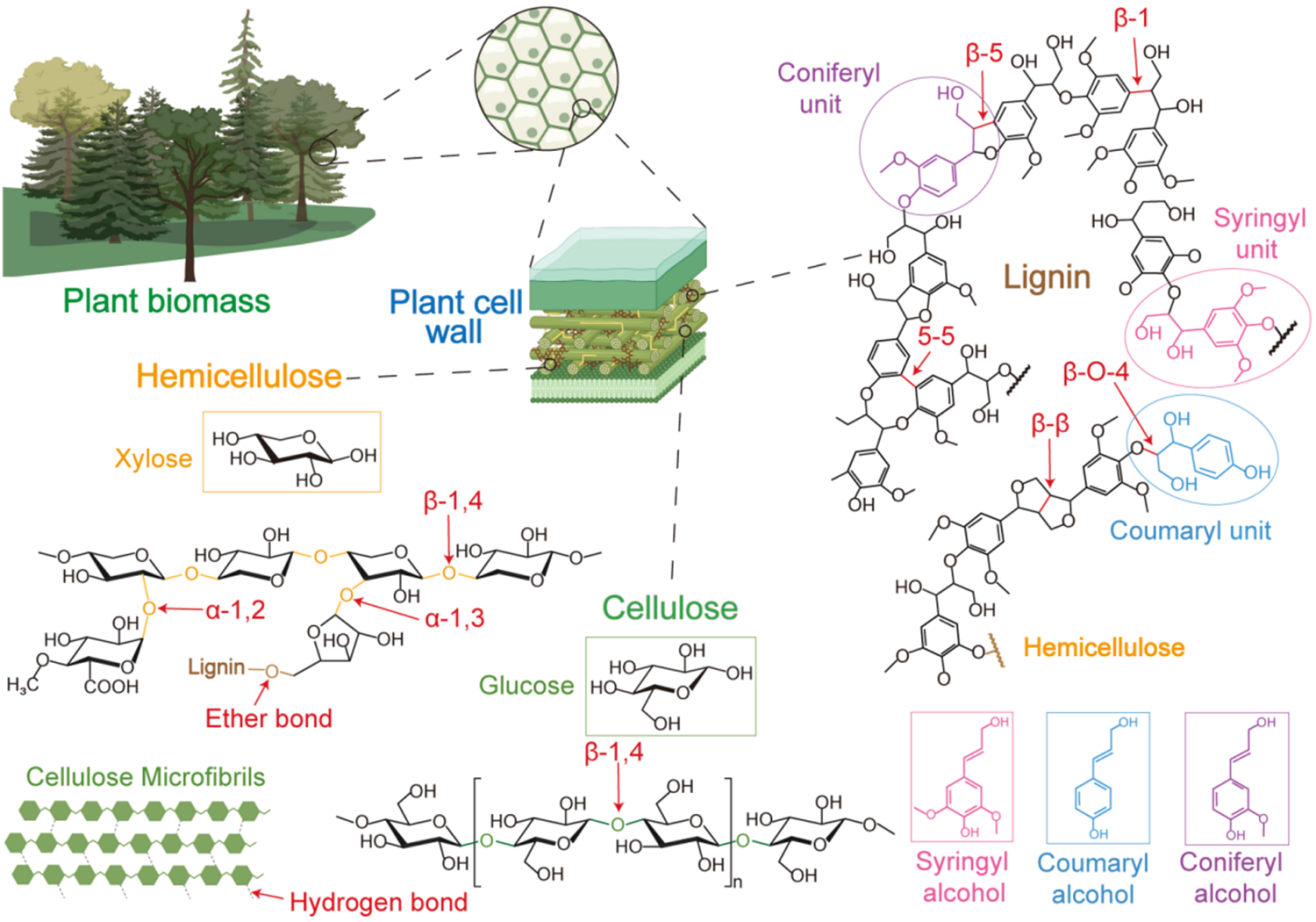
Schematic representation of lignocellulose structure and its major components. The complex and recalcitrant network is formed by polysaccharide polymers (cellulose and hemicellulose) and the aromatic polymer lignin, stabilized by various interactions. Cellulose is a linear homopolymer of d-glucose linked by β-1,4-glycosidic bonds. Hemicellulose (represented here as arabinoglucuronoxylan) has a backbone of β-1,4-linked d-xylose residues. The backbone is substituted with side chains including α-l-arabinofuranose and 4-*O*-methyl-α-d-glucuronic acid, typically attached via α-1,3 and α-1,2 linkages, respectively. Lignin is an aromatic heteropolymer derived from the oxidative coupling of three hydroxycinnamyl alcohols (monolignols): *p*-coumaryl, coniferyl, and sinapyl alcohols, which give rise to *p*-hydroxyphenyl (H), guaiacyl (G), and syringyl (S) units, respectively. The lignin structure depicted here is representative of grasses, which incorporate all three unit types. Covalent bonds (e.g., benzyl ether bonds shown in brown) between lignin and hemicellulose further contribute to the network’s stability and resistance to enzymatic deconstruction.

Lignin constitutes the major recalcitrant barrier in lignocellulose depolymerization, physically shielding structural polysaccharides from enzymatic attack while its degradation derivatives further inhibit cellulase activity. While hemicellulose contributes to recalcitrance by forming a interfacial layer where acetylation imposes steric and electrostatic constraints, lignin remains the principal barrier to enzymatic deconstruction.^11^ Efficient lignin depolymerization is essential for biomass valorization, yet it remains a significant technical bottleneck owing to the polymer’s structural heterogeneity and inherent physicochemical stability. Biorefineries target primarily cellulose and hemicelluloses by hydrolyzing these structural polysaccharides into monosaccharides, and then proceed through fermentation or integrated chemo-microbial processes to produce valuable end products, such as lactic acid and xylitol, or biofuels.^12^ The yield and kinetics of polysaccharide depolymerization govern the economic viability of industrial-scale lignocellulosic biorefining.^13^ Enzyme cocktail optimization and protein engineering are commonly used to improve catalytic efficiency and stability of the commercialized enzymes.^14^ The groundbreaking discovery of oxidative polysaccharide-degrading enzymes has expanded the enzymatic toolbox, offering powerful means to enhance the conversion of recalcitrant polysaccharides.^15,16^ As an alternative to these enzymatic strategies targeting mainly polysaccharides, the emerging “lignin-first” biorefining paradigm prioritizes the upstream fractionation of lignin polymers, thereby fundamentally bypassing the structural bottlenecks of the lignocellulose matrix. This proactive separation not only secures high-quality, native-like lignin streams for valorization but also significantly alleviates the steric exclusion and nonspecific adsorption of cellulases, effectively unleashing the full enzymatic potential for carbohydrate conversion.^17,18^

## 2 Co-Evolution of Plants and Biomass-Degrading Microorganisms

The evolutionary arms race between microorganisms and plant cell walls originated early in life’s history, driven by the microbial need to breach these recalcitrant barriers for nutrient acquisition or pathogenesis. Approximately 750 million years ago the algae *Streptophyte* emerged featuring a cell wall containing cellulose, xyloglucan, and pectin but lacking lignin.^19^ In response, microorganisms acquired powerful hydrolytic enzyme systems capable of efficiently degrading structural polysaccharides.^20^ During the late Ordovician to Silurian periods (approximately 420 million years ago), lignin was implemented as a key adaptation of plants to terrestrial environments.^21^ This increase in recalcitrance imposed strong selective pressures, forcing terrestrial microbial decomposers to rapidly evolve entirely new, predominantly oxidative, degradation strategies.

Within terrestrial ecosystems, filamentous fungi, particularly the *Agaricomycetes*, are the predominant lignocellulose decomposers.^22^ Their specific degradation strategies have evolutionarily diverged based on their ecological niches. For instance, while plant pathogenic fungi employ sophisticated mechanisms to circumvent host immune recognition,^23^ saprophytic wood-decaying fungi are entirely focused on dismantling recalcitrant barriers for nutrient acquisition. White-rot fungi evolved enzyme-driven paradigm for lignin depolymerization.^24^ Their secretomes boast an expansive arsenal of high-redox-potential oxidoreductases, notably laccases (EC 1.10.3.2) and lignin-active peroxidases (EC 1.11.1.-).^25,26^ Laccases utilize diffusible redox mediators to attack lignin polymers (up to +0.8 V vs SHE), while peroxidases exploit the oxidative power of hydrogen peroxide (H_2_O_2_) to directly cleave recalcitrant lignin macromolecules via potentials reaching +1.1 to +1.5 V.^27^ The arsenal of secreted enzymes involves a complete set of cellulases (e.g., endoglucanase, cellobiohydrolase, β-1,4-glucosidase), and lytic polysaccharide monooxygenases (LPMOs) as well (Table 1). In contrast, brown-rot fungi utilize a streamlined secretome,^28^ favoring a reactive oxygen species (ROS)-dependent mechanism^29^ that avoids the metabolic cost of high enzyme secretion.^30^ Transcriptomic analyses of the brown-rot fungus *Fibroporia radiculosa* reveal a significant upregulation of H_2_O_2_ **-**producing oxidases (e.g., alcohol oxidase) in the initial stages, indicating a prioritization of Fenton reactions to disrupt the lignin matrix, thereby increasing the accessibility of structural polysaccharides for subsequent enzymatic hydrolysis (Figure 2).^31^ H_2_O_2_ is generated at the tips of fungal hyphae together with Fe^2+^ to produce hydroxyl radicals. This process is operationally regulated by a pH-gradient, iron chelating species and radical scavenger systems.^32^ The oxidative repertoire is further expanded by the discovery of anaerobic fungi (*Neocallimastigomycetes*). These fungi deconstruct grass and hardwood lignin in anoxic niches by leveraging atypical, oxygen-independent pathways rather than classical oxidoreductases to cleave resilient linkages, such as β-aryl-ether (β-O-4) bonds.^33^ This ability to modify lignin without molecular oxygen represents a critical, uncharted frontier in the evolutionary adaptation of microbial biomass-degrading systems.

**Figure 2:**
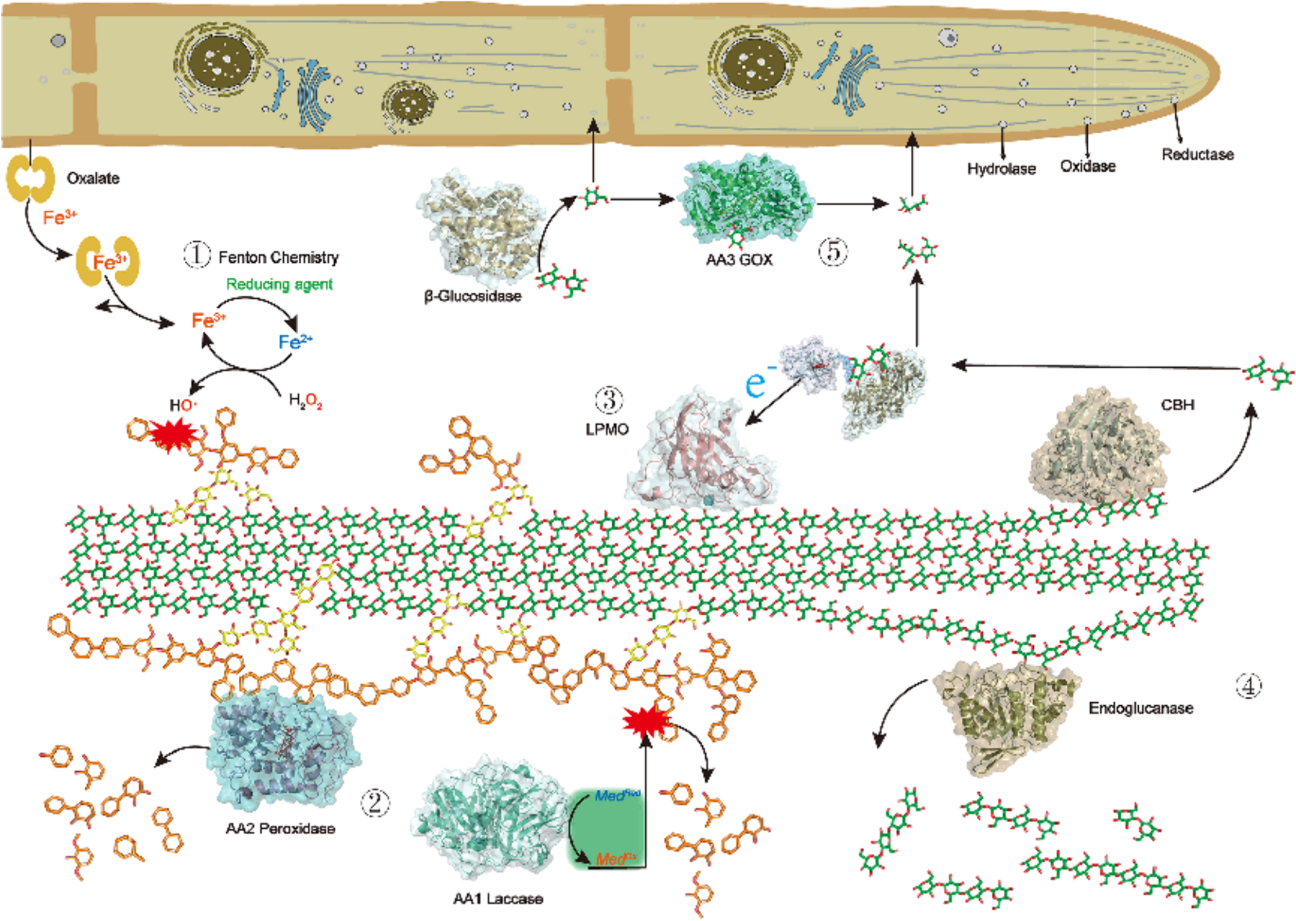
Functions of fungal enzymes during oxidative lignocellulose degradation. ① Fenton chemistry; ② Lignin-degrading enzymes; ③ Carbohydrate-cleaving LPMOs; ④ Carbohydrate-depolymerizing hydrolases; ⑤ H_2_O_2_ producing enzymes supporting the Fenton chemistry, peroxidases or LPMOs (GOX is shown as a representative example). Detailed enzymatic degradation network and biochemical synergism are shown in Figure 7.

**Table 1:** Discovered Lignin and Polysaccharide Degrading Oxidoreductases in Well-Characterized Fungi and Bacteria^c^

Organism	Type of Oxidoreductases	Cofactor	Substrate^a^	Reference
*Pleurotus* sp. (*Pleurotus ostreatus*, *Pleurotus eryngi*) (white rot fungus)	AA1 Laccase	Copper	ABTS	^40^
	AA2 Dye-decolorizing peroxidase	Heme	Zearalenone, 2,6-DMP, RB5, RB4, RB19, Mn^2+^	^41,42^
	AA2 Versatile peroxidase	Heme	Mn^2+^, ABTS, RB5, 2.6-DMP, VA	^43^
	AA2Manganese peroxidase	Heme	Mn^2+^, ABTS, 2,6-DMP	^43^
	AA3/AA5 Aryl-alcohol oxidase	FAD	VA	^44,45^
	AA9 lytic polysaccharide monooxygenase	Copper	n.d.	^46^
*Trametes* sp. (*Trametes versicolor*, *Trametes hirsuta*, *Trametes trogii*) (white rot fungus)	AA1 Laccase	Copper	n.d.	^47^
	AA2 Dye-decolorizing peroxidase	Heme	ABTS, 2,6-DMP, RB5, RB19, Mn^2+^	^48^
	AA2 Versatile peroxidase	Heme	Mn^2+^, VA, RB5, RB19, DMP, ABTS	^48,49^
	AA2Manganese peroxidase	Heme	Mn^2+^	^50^
	AA2 Lignin Peroxidase	Heme	n.d.	^51^
	AA3–1 Cellobiose dehydrogenase	FAD/Heme	Cellobiose, Lactose, Xylobiose, Glucose, Maltose	^52^
	AA3 Pyranose oxidase	FAD	Glucose	^53^
	AA5 Glyoxal oxidase	Copper	n.d.	^54^
	AA14 lytic polysaccharide monooxygenase	Copper	Xylan^b^	^55^
*Phanerochaete* sp. (*Phanerochaete chrysosporium*, *Phanerochaete sordida*) (white rot fungus)	AA2Manganese peroxidase	Heme	n.d.	^56,57^
	AA2 Lignin Peroxidase	Heme	n.d.	^56^
	AA3–1 Cellobiose dehydrogenase	FAD/Heme	Cellobiose, Lactose, Glucose	^58,59^
	AA5 Glyoxal oxidase	Copper	Methylglyoxal	^60^
	AA9 lytic polysaccharide monooxygenase	Copper	n.d.	^58^
*Gloeophyllum trabeum* (Brown rot fungus)	AA1 Laccase	Copper	n.d.	^61^
	AA3/AA5 Alcohol oxidase	FAD	n.d.	^61^
	AA3 Pyranose oxidase	FAD	n.d.	^61^
	AA6 P-benzoquinone reductase	NAD(P)H	n.d.	^61^
	AA9 lytic polysaccharide monooxygenase	Copper	Cellulose, Xyloglucan	^61,62^
*Postia placenta* (Brown rot fungus)	AA1 Laccase	Copper	n.d.	^63^
	AA3 Glucose oxidase	FAD	n.d.	^29^
	AA3 Alcohol oxidase	FAD	n.d.	^29^
	AA3 Aryl-alcohol oxidase	FAD	n.d.	^29^
	AA5 Glyoxal oxidase	Copper	n.d.	^29^
	Polyphenol oxidase	Copper	n.d.	^63^
*Neurospora* sp. (*Neurospora discrete*, *Neurospora crassa*) (Phytopathogenic fungus)	AA2 Versatile peroxidase	Heme	RB5	^64^
	AA3–1 Cellobiose dehydrogenase	FAD/Heme	Cellobiose, Lactose, Xylobiose, Glucose, Maltose	^65^
	Polyphenol oxidase	Copper	Pyrocatechol	^64^
	AA9 lytic polysaccharide monooxygenase	Copper	PASC, Tamarind xyloglucan, Konjac glucomannan, Cellopentaose	^66,67^
*Thermothelomyces thermophila* (Phytopathogenic fungus)	AA1 Laccase	Copper	ABTS, Catechol, 2,6-DMP, Pyrogallol, Guaiacol	^68,69^
	AA3–1 Cellobiose dehydrogenase	FAD/Heme	n.d.	^70^
	AA3 Aryl-alcohol oxidase	FAD	VA, Cinnamyl alcohol, Anisyl alcohol	^71^
	AA5 Glyoxal oxidase	Copper	n.d.	^70^
	AA9 lytic polysaccharide monooxygenase	Copper	n.d.	^70^
	AA16 lytic polysaccharide monooxygenase	Copper	Beechwood xylan, Wheat Arabinoxylan	^72^
*Aspergillus* sp. (*Aspergillus niger*, *Aspergillus terreus*, *Aspergillus fumigatus*, *Aspergillus aculeatus*, *Aspergillus oryzae*) (Phytopathogenic fungus)	AA1 Laccase	Copper	n.d.	^73^
	AA3 Glucose oxidase	FAD	n.d.	^74^
	AA3 Alcohol oxidase	FAD	n.d.	^75^
	AA3 Glucose dehydrogenase	FAD	Glucose	^76^
	AA9 lytic polysaccharide monooxygenase	Copper	PASC, Beechwood Xylan, Xyloglucan	^77^
	AA16 lytic polysaccharide monooxygenase	Copper	PASC	^78^
	AA17 lytic polysaccharide monooxygenase	Copper	Guaiacol, 2,6-DMP, 3,4-Dimethoxy benzyl alcohol	^79^
*Streptomyces* sp. (*Streptomyces lividans*, *Streptomyces coelicolor*) (Actinomycete bacterium)	AA1 Laccase	Copper	ABTS	^80^
	AA2 Dye-decolorizing peroxidase	Heme	n.d.	^81^
	AA2 Lignin Peroxidase	Heme	VA	^80^
	AA10 lytic polysaccharide monooxygenase	Copper	PASC, β-chitin	^82,83^
*Pseudomonas fluorescens* (Gammaproteobacteria)	AA2 Dye-decolorizing peroxidase	Heme	ABTS, Mn^2+^, Alkali Kraft lignin	^84^
*Acinetobacter* sp. (*Acinetobacter radioresistans*, *Acinetobacter baumannii*) (Gammaproteobacteria)	AA1 Laccase	Copper	ABTS, 2,6-DMP	^85^
	AA2 Dye-decolorizing peroxidase	Heme	ABTS	^86^

a Substrate: Refers to all natural and non-natural substrates confirmed by experimental assays. Instances identified only via transcriptomic analysis without experimental validation are marked as n.d.

b AA14 activity on xylan was reported though not confirmed in later studies.^87^

c Abbreviations FAD: Flavin adenine dinucleotide; ABTS: 2′-amino-di (3-ethylbenzthiazoline sulfonicacid-6) ammonium salt 2; 2,6-DMP: 2,6-Dimethylphenol; RB5: Relative Black 5; RB19: Relative Blue 19; RB4: Relative Blue 4; Mn2+: Manganese­(II) ion; PASC: Phosphoric acid swollen cellulose; VA: Veratryl alcohol.

Beyond fungi, prokaryotes also represent potent decomposers of plant cell walls, renowned for their remarkable genetic diversity and possession of highly adaptable enzymes. While aerobic bacteria often deploy secreted catalytic networks analogous to fungal systems, utilizing dye-decolorizing peroxidases (DyPs) in tandem with quinone reductases and superoxide dismutases to induce Fenton-like lignin oxidation,^34,35^ anaerobic microorganisms showcase remarkable spatial and metabolic adaptations. Anaerobic cellulolytic bacteria, such as *Clostridium*, evolved cellulosomes: scaffold-bound multienzyme complexes that physically coordinate the sequential hydrolysis of structural polysaccharides to maximize degradation efficiency.^36,37^ Additionally, the genomes of anaerobic cellulolytic bacteria also harbor oxidative enzymes including LPMOs.^38^ But, direct experimental validation remains limited. Besides, the rumen of herbivores and the gut of termites are also key symbiotic ecological niches and functional evolutionary units for lignocellulose-degrading microbial communities.^39^ These highly specialized microenvironments host intricate consortia where bacteria, fungi, and protozoa operate in metabolic syntropy, leveraging compartmentalized enzymatic repertoires to achieve the rapid degradation of complex biomass.

Historically, the microbial degradation of exposed structural polysaccharides was believed to depend exclusively on glycoside hydrolases. However, this perception was fundamentally overturned in 2010 with the discovery of LPMOs.^16^ Initially, research focused on the ability of (LPMOs; EC 1.14.99.-) to cleave crystalline polysaccharides such as cellulose, chitin and starch. This cleavage promotes the swelling and decrystallization of cellulose microfibrils, thereby facilitating their subsequent, more efficient depolymerization by glycoside hydrolases.^88^ Subsequently, interest shifted to the catalytic versatility of LPMOs, which are now known to act not only on cellulose, chitin, and starch but also on various hemicelluloses. Accordingly, LPMOs are currently classified within nine Auxiliary Activity (AA) families in the Carbohydrate-Active enZymes database (CAZy).^89^ The catalytic efficacy of LPMOs relies entirely on a coevolved network of electron donors, most notably cellobiose dehydrogenase (CDH; EC 1.1.99.18), cellooligosaccharide dehydrogenase (CelDH; EC 1.1.99.-) and H_2_O_2_-generating auxiliary enzymes.^15,90^ Consequently, an intricate biotic and abiotic oxidative degradation network, comprising LPMOs, dynamic cosubstrate regulators, and nonenzymatic quinone cycling,^91,92^ has been classified into various AA families. The remarkable diversity of these discovered lignin- and polysaccharide-degrading oxidoreductases across well-characterized fungi and bacteria is systematically summarized in Table 1.

Since the seminal reviews by Bissaro et al. and Frommhagen et al. in 2018,^91,93^ the landscape of redox enzymes in biomass conversion has evolved rapidly. Beyond the continued elucidation of LPMO catalytic mechanisms, the field has been enriched by the discovery of new AA families and novel oxidative enzymes. This experimental insights are increasingly supported by advancements in deep learning-based structural prediction, which facilitate the generation of accurate protein models for previously uncharacterized enzymes. In this review, we bridge these recent experimental as well as computational studies by integrating structural bioinformatics with mechanistic insights. We provide a systematic summary of the oxidoreductase networks involved in lignocellulose degradation, offering a refined perspective on their evolutionary adaptations and future biotechnological applications.

## 3 Key Enzymes Driving Oxidative Lignin Depolymerization

In natural ecosystems, lignin depolymerization is a complex, multistep process involving diverse enzyme systems and chemical species. This process can be broadly divided into two stages: the initial depolymerization of lignin and the subsequent transformation of aromatic compounds. The initial step involves oxidoreductases such as laccases and peroxidases, which efficiently cleave the β-O-4 linkages, the most abundant structural motifs in lignin.^94,95^ While these enzymes can also initiate the modification of more recalcitrant linkages, such as β-5 and 5-5, complete cleavage usually requires extensive oxidative processes or synergy with auxiliary enzyme systems.^96,97^ These reactions generate phenylpropane monomeric structures; however, the radical-mediated mechanism of these enzymes leads to the intermediate repolymerization, which potentially competes with depolymerization and reduces the yield of soluble products.^96^ The liberated monomeric structures are further enzymatically transformed into compounds like *p*-coumaric acid, vanillic acid, and *p*-hydroxybenzoic acid via decarboxylation, double-bond cleavage, side-chain fragmentation, ether cleavage, and oxidation.^98^ Guided by the metabolic requirements of the cell, these monomers are subsequently imported into cells and catabolized. For instance, *p*-coumaric acid and vanillic acid undergo enzymatic transformations, including demethylation and hydroxylation, to form diphenolic intermediates such as catechol derivatives.^99^ These diphenols are then subjected to aromatic ring cleavage catalyzed by dioxygenases.^100^ Ultimately, the aromatic ring is cleaved, and the resulting intermediates are funneled into the tricarboxylic acid (TCA) cycle through distinct pathways.^101,102^

The depolymerization of polymeric lignin is believed to be the rate-limiting step in the deconstruction of plant cell walls under controlled conditions. This critical step is tightly regulated and primarily driven by laccases and peroxidases in white rot fungi, which oxidize lignin to generate phenoxy radicals that mediate further depolymerization reactions, whereas brown-rot fungi have also evolved a complementary approach using Fenton chemistry.^103^ By oxidizing aromatic side chains and other degradation products, auxiliary oxidases such as aryl-alcohol oxidase (AAO), methanol oxidase (MOX), and glycolate oxidase, as well as copper radical oxidases establish H_2_O_2_ as a pivotal linking node within the oxidative enzyme network, enabling synergistic activity of lignin-active peroxidases.^104^ This section will focus on the principal lignin-degrading enzymes, namely laccase and peroxidase, emphasizing their structural characterization and central roles in lignin depolymerization.

### 3.1 Laccase

Laccase (EC 1.10.3.2) as a multicopper oxidase, harnesses molecular oxygen to generate phenolic radicals, playing a key role in the oxidative deconstruction of lignin. The evolutionary origin of multicopper oxidases is supposed to be a single-domain cupredoxin protein, like rusticyanin.^105^ Two-domain multicopper oxidases probably serve as the evolutionary intermediates, ultimately evolving into the three-domain form.^106,107^ Fungal laccase is a three-domain multicopper oxidases (β-trefoil) in a single polypeptide chain, with copper ions coordinated at the interface of these domains. The bacterial-derived “small laccases”, with one or two domains, form distinct clusters in phylogenetic analysis due to cross-lineage diversification.^105^ It is a polyphenol oxidase (PPO) with a broad substrate spectrum, which belongs to the AA1 family of the CAZy database and is currently classified into three subfamilies based on sequence similarity (Figure 3; Figure S1).^108^ Laccase typically contains a trinuclear Cu cluster and the type 1 copper at the T1 site gives its characteristic blue color. The histidine residues coordinating four copper ions are highly conserved across the three subfamilies (Figure 3).

**Figure 3:**
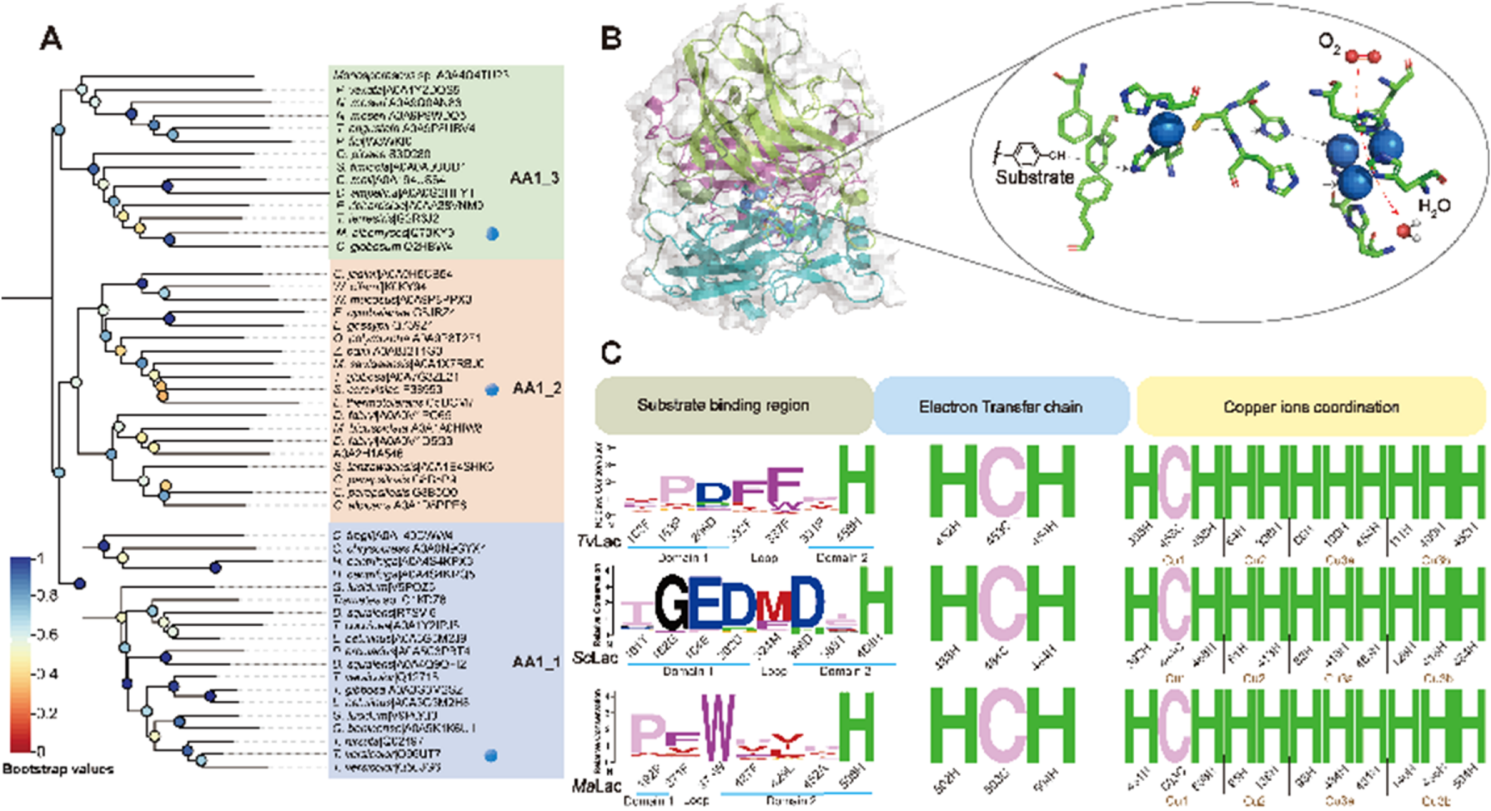
Laccase evolutionary relationship, active site architecture and sequence logo. (A) Phylogenetic tree of laccases. Subfamily 1 (AA1_1): blue; Subfamily 2 (AA1_2): orange; Subfamily 3 (AA1_3): green. Blue points in panel A indicate the representative sequences selected for sequence logo generation in panel C. (B) Schematic diagram of the laccase catalytic cycle, depicting the oxidation of phenolic substrates and the concomitant four-electron reduction of molecular oxygen to water (PDB: 1KYA). (C) Sequence logo of key residues across different subfamilies. All sequences used for sequence logo generation are provided in a separate FASTA file. Detailed procedures for the bioinformatic analysis are provided in Figure S1.

In the catalytic reaction of laccase, a phenolic molecule binds to the active site and an electron is abstracted from the T1Cu coordinating histidine to the T1 copper. The electron is then further passed through a highly conserved histidine-cysteine-histidine (HCH) electron transfer chain to the trinuclear copper cluster, where oxygen is reduced to water by accepting four subsequent electrons.^109,110^ A complete catalytic cycle requires continuous oxidation of four substrate molecules to reduce molecular oxygen. The three subfamilies possess distinct substrate-binding regions, reflecting their different functional tendencies. Subfamily 1 and 3 are composed of mostly hydrophobic amino acids consistent with lignin’s hydrophobicity, while subfamily 2 has highly conserved negatively charged amino acids (Figure 3), which has been reported with ferroxidase activity.^111^ The high catalytic activity of fungal laccases toward small-molecule mediators enables efficient lignin degradation when operating as part of a laccase-mediator system (LMS), thereby boosting the biomass-degrading capacity of fungi.^26^ This property is markedly distinct from plant laccases and small laccases.

Laccases exhibit oxidation potentials of +0.45 up to +0.8 V vs SHE, enabling them to directly oxidize phenolic hydroxyl groups in lignin to generate phenoxy radicals. However, due to the low abundance of free phenolic hydroxyl groups in the native lignin polymer, laccases circumvent this limitation by prioritizing the oxidation of diffusible mediators (e.g., lignin-derived syringyl and guaiacyl compounds).^112,113^ These radicals subsequently undergo secondary, nonenzymatic reactions, that promote further depolymerization of lignin macromolecules, ultimately yielding a diverse array of aromatic compounds, including monophenols, diphenols, polyphenols, aminophenols, and methoxyphenols.^114^ Due to the radical-based reaction mechanism, laccases are regarded to have broad substrate promiscuity. However, emerging experimental evidence points to the potential presence of substrate specificity. The primary oxidation by laccases involves the consecutive oxidation of four substrate molecules, which indicates that substrate dissociation also plays a crucial role in reaction efficiency.^115^ Lignin consists of three structural units, distinguished by their methoxy group content (Figure 1). Methoxy-rich units may form nonessential hydrogen bonds and impede product dissociation, thereby reducing catalytic turnover. Simulation data and kinetic analyses of laccases support this hypothesis. *Trametes versicolor* laccase displays higher interaction energy when binding to 2,6-dimethoxyphenol compared to guaiacol—two structural analogs differing in methoxy group content.^116^ Similarly, laccase from *Sordaria macrospora* exhibites a higher *K*_M_ but a significantly higher *k*_cat_/*K*_M_ for guaiacol, indicating that tighter substrate binding may be detrimental to catalytic efficiency.^117^ This inverse relationship between binding affinity and turnover efficiency has been observed in other laccases as well.

The contribution of laccases to lignin depolymerization is increasingly recognized as a sophisticated strategy involving self-generated mediators and synergistic enzyme networks.^97^ A study has revealed that laccases can utilize small phenolic intermediates generated during lignin degradation as mediators, thereby facilitating further depolymerization of lignin.^118^ This indicates that in nature, laccase may adopt such intermediates as the primary strategy for participating in lignin depolymerization, in order to enhance lignin degradation. Studies employing both laccases and peroxidases for lignin treatment have highlighted the pivotal role of laccase to attack lignin.^119,120^ Laccase utilizes environmental O_2_ as an oxidant, producing highly reactive radicals and intermediates through the oxidative half-reaction. These radicals can subsequently serve as oxidants for polysaccharide-oxidizing enzymes, potentially contributing to the intricate network of oxidative reactions. However, the radical-based reaction mechanism of laccases can lead to uncontrolled product repolymerization.^121,122^ Studies have shown that the presence of multiple enzymes enhances lignin depolymerization compared to single-enzyme system, emphasizing the advantage of a synergistic multienzyme approach.^123^

### 3.2 Lignin Active Peroxidase

Peroxidases involved in lignin degradation include lignin peroxidase (LiP), manganese peroxidase (MnP), versatile peroxidase (VP), and DyP.^103^ They are grouped in the AA2 family in the CAZy database and require hydrogen peroxide (H_2_O_2_) to initiate catalysis and generate products such as phenols and diphenols. The RedOxiBase database classifies LiP, MnP, and VP as Class II heme peroxidases, whereas DyP belongs to a distinct family with limited sequence and structural similarity to Class II heme peroxidases.^124^ DyPs can be further divided into four subfamilies: A, B, C, and D, which is visible in the phylogenetic tree (Figure 4). LiP exhibits a high redox potential of +1.2 V vs SHE, allowing it to directly oxidize nonphenolic aromatic compounds.^125^ MnP has a Mn­(II) binding site additionally to the heme-containing active site and catalyzes substrate oxidation via an indirect mechanism. At first, Mn­(II) oxidizes to Mn­(III), which subsequently acts as a diffusible oxidant targeting phenolic substrates but not nonphenolic ones.^126^ MnP is the most abundant ligninolytic peroxidase found in the secretome of white-rot fungi.^91^ VP, with an exceptionally high redox potential of +1.4 V vs SHE, integrates the functions of LiP and MnP, enabling it to oxidize both phenolic and nonphenolic compounds.^127^ Notably, bacterial-derived DyP exhibits dual catalytic functionality, combining radical-mediated oxidative cleavage (e.g., Cα-Cβ bond scission) with hydrolytic activity (β-aryl ether bond hydrolysis) on lignin model compounds.^128^ Importantly, DyP-type peroxidases have also been shown to have activity for oxidation of polymeric lignin, such as alkali Kraft lignin.^129,130^ However, its precise catalytic mechanism remains unclear.

**Figure 4:**
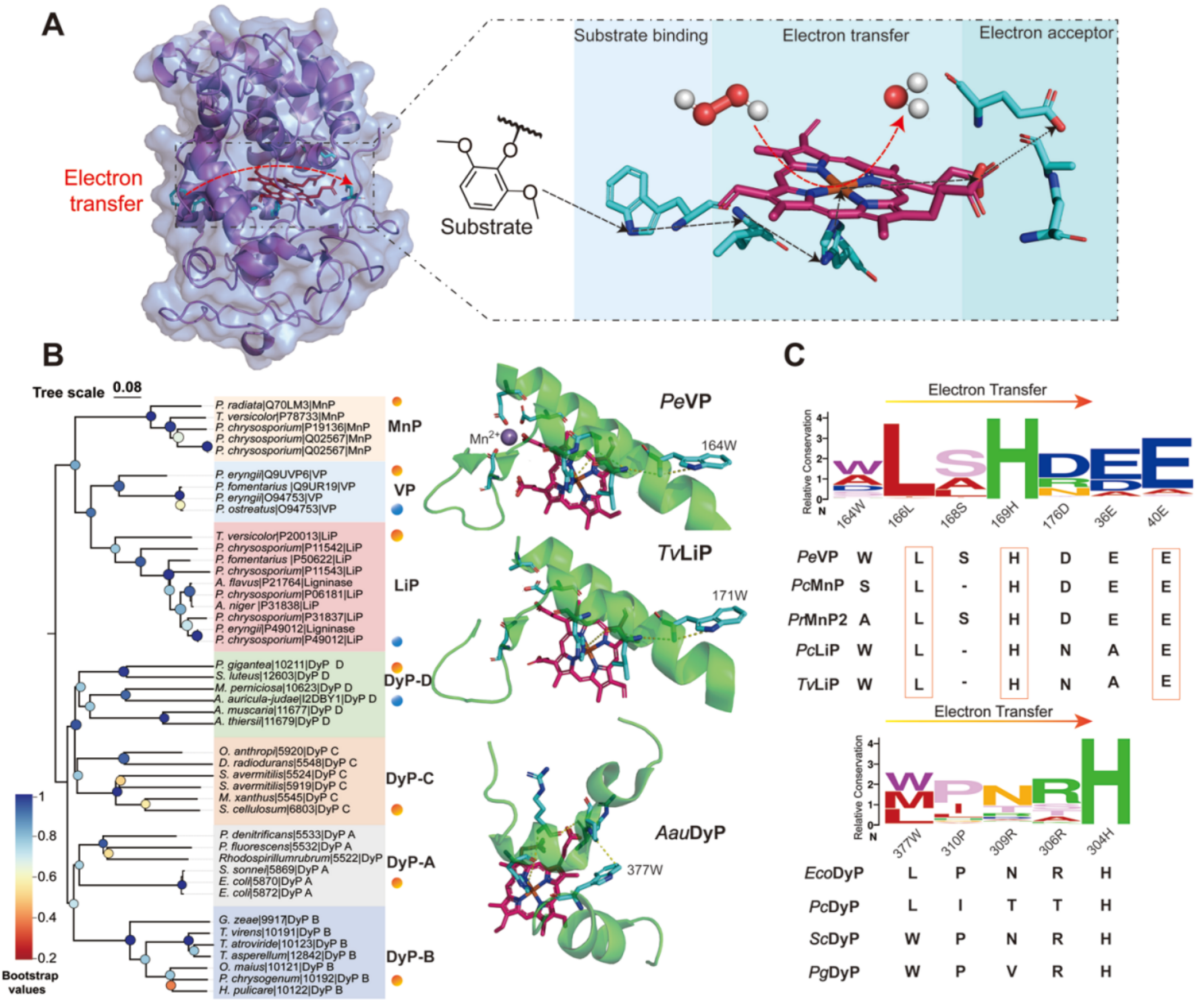
Evolutionary relationship, active site architecture and sequence logo of the electron transfer chain of lignin active peroxidases. (A) Schematic diagram of the catalytic mechanism of the lignin active peroxidases. (B) Phylogenetic tree of AA2 peroxidases and schematics of electron transfer chains across different subfamilies. LiP: lignin peroxidase (PDB: 1LIP); MnP: manganese peroxidase; VP: versatile peroxidase (PDB: 2BOQ); DyP: dye decolorization peroxidase (PDB: 4W7J). Orange points in this panel indicate the representative sequences selected for depicting the electron transfer pathways. (C) Sequence logo of key residues constituting the electron transfer pathways. Blue points in panel B indicate the representative sequences selected for sequence logo generation in panel C. Extended sequence logo of active site architecture and cofactor coordination are provided in Figure S2. Sequences used for sequence logo generation are provided in a separate FASTA file.

The molecular structure of ligninolytic peroxidases features a heme cofactor within an internal cavity serving as the active center, along with two functional channels. The main channel, conserved across all four enzyme types, facilitates the entry of H_2_O_2_ to activate the enzyme. The second channel, located at the propionate group of the heme cofactor, is where Mn^2+^ is oxidized to Mn^3+^ in MnP and VP.^131^ MnP and VP feature Mn^2+^ binding sites formed primarily by glutamate (Glu) and aspartate (Asp) residues. The carboxylate groups of these amino acids, along with the heme propionate, bind Mn^2+^ and enable subsequent electron transfer to the cofactor heme.^132^ The narrow channel limits the ability of these peroxidases to oxidize lignin macromolecules directly. As an alternative, long-range electron transfer (LRET) from the substrate to the cofactor or Mn^2+^ has been proposed. In high-redox-potential peroxidases (e.g., LiP, VP, and DyP), a surface exposed tryptophan residue was reported the function as oxidation sites for the aromatics, dyes, and lignin polymers.^133^ This tryptophan residue was reported to abstract electrons from the substrate and to initiate the LRET.^134^ However, sequence conservation analysis (Figure 4, Figure S2) reveals that this tryptophan residue is strongly conserved within specific subfamilies such as lignin peroxidases (LiP), shows much lower conservation across the broader family of high-redox-potential peroxidases (e.g., when comparing LiP, VP, and DyP). This suggests the existence of alternative electron transfer pathways, which necessitates further experimental validation. The LRET chain often aligns with an α-helix, except in DyP (Figure 4). Computational modeling and site-directed mutagenesis studies revealed that LRET in DyP involves two short α-helices facilitating electron transfer from substrates to the cofactor heme.^135^ In bacteria, a recent study showed a DyP-based catalytic network involved in lignin degradation used by *Pseudomonas sp.*. Supported by auxiliary enzymes such as quinone reductases, nitroreductases, and superoxide dismutase, this bacterial oxidative system promotes quinone redox cycling and Fenton chemistry to enhance lignin breakdown.^35^

During lignin degradation, laccase-mediated reactions generate free radicals and other highly reactive intermediates first. Then intermediates such as acetosyringone act as mediators for laccases and peroxidases, enhancing enzyme synergy and inhibiting radical repolymerization.^119^ In contrast to laccases, peroxidases leverage their high redox potential to directly degrade nonphenolic portions of lignin. Products of peroxidase-mediated lignin degradation, such as guaiacol, vanillyl alcohol, and eugenol, are often resistant to further degradation by peroxidases. Laccases, however, can utilize these phenolic compounds to generate phenoxy radicals, leading to ring-opening reactions in the presence of oxygen.^120^ These observations underscore the importance of multienzyme systems for efficient lignin depolymerization and indeed laccases and peroxidases are commonly cosecreted in natural systems. Other studies have indicated that lignin degradation products generated by lignin-active peroxidases can enhance LPMO activity when both enzymes are coexpressed.^136,137^ However, peroxidases also compete with LPMOs for H_2_O_2_.^91^ The complex interaction of theses enzymes, supporting/promoting enzymatic activities or competing for substrates, is difficult to delineate during *in vitro* experiments, suggesting a delicate, kinetic balance of oxidoreductase-based degradation systems in nature.

## 4 Oxidative Degradation of Polysaccharides

LPMO-mediated oxidative cleavage of the glycosidic bonds has garnered significant attention due to their unique catalytic diversity and mechanism. LPMOs have been widely recognized as key components of enzyme cocktails employed in the industrial processing.^138,139^ LPMOs and their auxiliary enzymes form a powerful oxidative degradation network, significantly enhancing the efficiency of conventional hydrolases.^140,141^ Besides LPMOs, the nonspecific radical attack of cellulose and hemicelluloses by hydroxyl radicals (^•^OH) is the known nonhydrolytic depolymerization mechanism. In addition to these two already known oxidative pathways, the recently discovered enzyme CelOCE makes use of an exoacting oxidative mechanism releasing cellobionic acid units.^142^

### 4.1 LPMO Classification and Structural Characterization

LPMOs have evolved an active site that coordinates the catalytic copper ion to generate highly reactive oxygen species, which serve as powerful agents to specifically target and cleave glycosidic bonds with high energy barrier (397–440 kJ/mol).^143,144^ Several recent reviews have provided detailed discussions on the substrate diversity and catalytic mechanism of all LPMOs,^91,145–147^ so this section will only focus on the four LPMO families (AA9, AA10, AA14, and AA15) that exhibit activity on cellulose or hemicellulose, especially the architecture of the active sites, the substrate-binding regions, and the copper-ligating amino acids.

Crystal structures reveal that LPMOs generally have a solvent-exposed active site, which contains two conserved histidine residues mostly within a flat surface (Figure 5). These histidine residues coordinate the exposed Cu^2+^ ion together with the N-terminal amino group, forming the so-called “histidine brace”, a feature fully conserved across the LPMO superfamily.^148^ This coordination motif confers a strong Cu­(II) binding affinity, a property established early in LPMO research through biophysical methods such as NMR and isothermal titration calorimetry.^149,150^ Recent work by Munzone et al. (2024) has further refined it by employing ligand competition assays to provide a rigorous quantitative framework for understanding these binding dynamics.^151^ Additionally, there is an axial residue located deeply in the protein, which is always a highly conserved aromatic hydrophobic amino acid (Figure 5). In AA9 and AA14, this axial residue is typically tyrosine, while in AA10 and AA15 is phenylalanine. Mutagenesis and electrochemical studies have demonstrated that the chemical nature and distance of this axial side chain from the copper ion are critical for fine-tuning the redox potential and the overall reactivity of the enzyme.^152,153^ However, most mechanistic insights to date are derived from the AA9 and AA10 families, leaving the functional nuances of other LPMO families as an area for further investigation.

**Figure 5:**
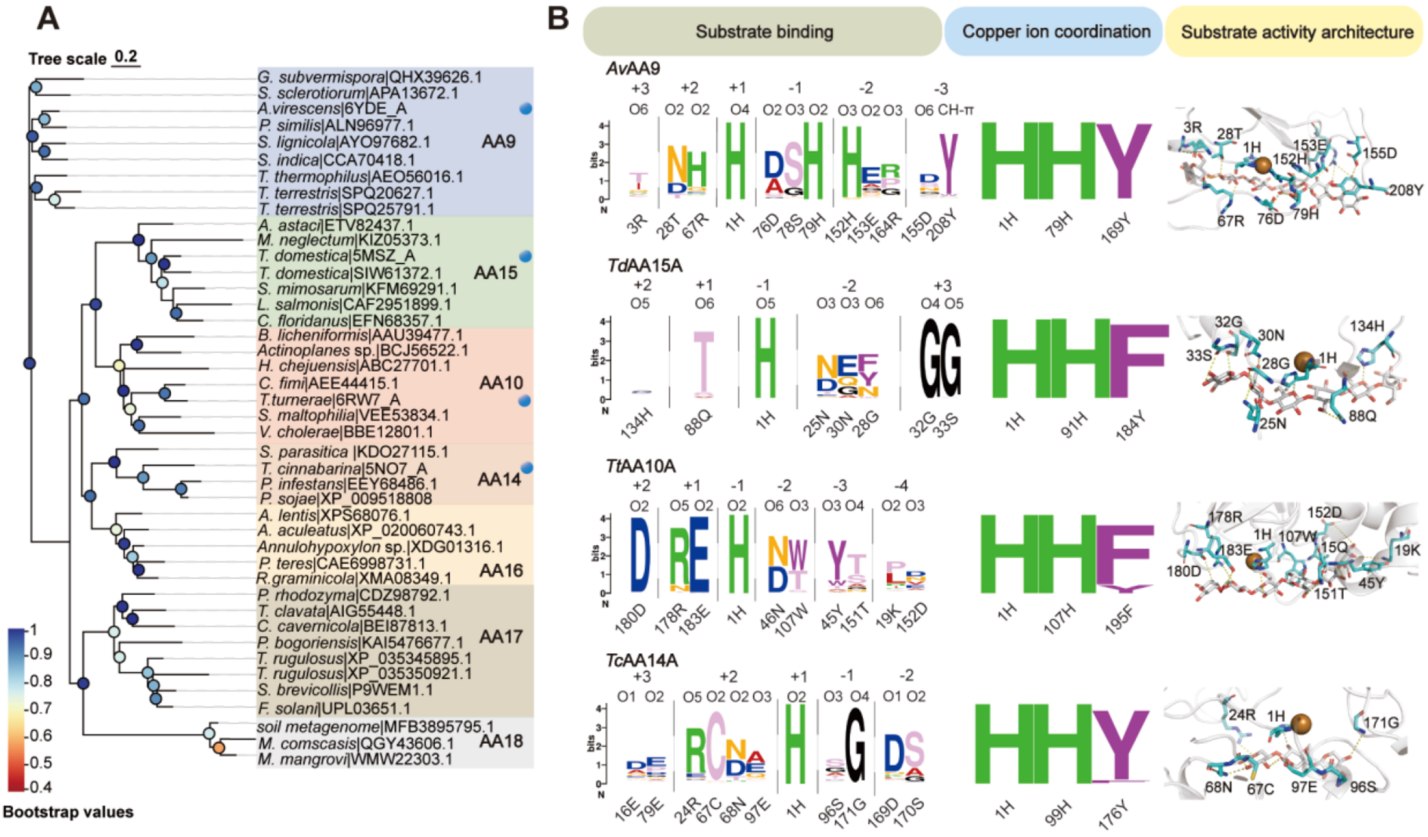
Evolutionary relationship, active site architecture and sequence logo of LPMO families. (A) Phylogenetic tree of the LPMO families. Blue points in this panel indicate the representative sequences selected for sequence logo generation in panel B. (B) Sequence logos and substrate-binding architectures of four lignocellulose-active LPMO families. Substrates used for modeling: cellohexaose (AA9, AA10, AA15) or xylan (AA14). Source PDB IDs: *Av*AA9 (6YDE), *Tt*AA10A (6RW7), *Tc*AA14A (5NO7), and *Td*AA15A (5MSZ). Sequences used for sequence logo generation are provided in a separate FASTA file.

Beyond the catalytic coordination, LPMOs utilize variable and flexible loop regions for substrate binding, where hydrogen bonding networks play a critical role in positioning the substrate.^154,155^ Although LPMOs typically interact with crystalline substrate surfaces rather than sequestering individual polysaccharide chains, substrate recognition is mediated by the coordination of specific sugar monomers with conserved residues at the binding interface—a well-established paradigm in carbohydrate-active enzymes (CAZymes).^155,156^ Aligning with this principle, our structural analyses indicate that LPMO binding regions frequently utilize polar or charged residues, such as aspartate, arginine, and histidine, to stabilize the enzyme–substrate complex through targeted interactions (Figure 5). Particularly, the histidine at the N-terminal not only coordinates the Cu^2+^ ion, but also interacts with the sugar ring at position +1 or −1 (Figure 5).^157,158^ The sequence comparison and structural analysis shown here indicates, that this pattern is observed across the four families discussed here (AA9, AA10, AA14, and AA15). This interaction is crucial for correct positioning of the substrate and for regulating the redox reactions, thereby preventing deviations from the intended catalytic pathway.^147,159^

A closer examination of the substrate-binding interface reveals a mosaic architecture comprising both highly conserved and variable residues (Figure 5). We hypothesize that the conserved “hotspots” underpin essential interactions required for substrate recognition, whereas the more variable positions may provide the plasticity needed to accommodate diverse polysaccharide architectures. This structural heterogeneity likely reflects functional adaptation, allowing different LPMO families to fine-tune their affinity for specific substrates, such as crystalline cellulose or various chitin polymorphs. The critical role of this surface composition is underscored by experimental evidence; for instance, mutagenesis of surface residues in AA10 LPMOs has been shown to directly dictate substrate binding specificity, catalytic regioselectivity (C1 vs C4 oxidation), as well as thermal stability.^160^

This structural determination of regioselectivity is further modulated by the physicochemical properties of the substrate.^161,162^ Although many cellulolytic LPMOs display strict regioselectivity for either the C1 or C4 carbon atoms, structural analyses reveal that both positions are in close proximity to the copper site. This proximity suggests that even subtle shifts in substrate positioning within the binding groove can pivot the oxidative outcome.^160^ Although the details are still being elucidated, some studies highlight that LPMO regioselectivity is determined by both enzyme architecture and as well as by the physical properties of the substrate.^163^ Notably, the crystallinity of cellulose is emerging as a significant extrinsic factor modulating the ratio of C1 and C4 oxidative product.^164^ Evidence suggests that for certain C1/C4-oxidizing AA9 LPMOs, the distribution of oxidized products can shift depending on whether the enzyme acts on highly crystalline versus amorphous regions of the cellulose.^165^ This product distribution is likely governed by the complex interplay between the enzyme’s flat carbohydrate-binding surface and the varying accessibility of the substrate surface topology. Recognizing this substrate-mediated modulation is essential for understanding how LPMOs optimize biomass deconstruction across heterogeneous cellulose surfaces.

### 4.2 Catalytic Mechanism and Cosubstrate of LPMO

Initially, LPMOs were classified as monooxygenases, however in recent years, a H_2_O_2_-dependent catalytic mechanism has gained broad acceptance and is supported by substantial experimental evidence.^166–169^ The catalytic reaction begins with the reduction of LPMO-Cu­(II) to Cu­(I). After this priming reduction, the enzyme interacts with H_2_O_2_ in a substrate-dependent manner. The substrate-enzyme-H_2_O_2_ interaction leads to substrate hydroxylation, the release of a water molecule, and the regeneration of LPMO-Cu­(I), which enables the initiation of a new catalytic cycle.^147^ Kittl et al. demonstrated that, in the presence of a reducing agent without substrate, LPMOs can function as oxidases, reducing O_2_ to generate H_2_O_2_.^170^ Quantum chemical calculations support that the proximal O atom of Cu­(II)–OOH abstracts a hydrogen atom from a small-molecule reductant (such as ascorbic acid), leading to the formation of LPMO–Cu­(I) and H_2_O_2_.^171,172^ This reveals an alternative oxidase pathway where LPMOs can generate H_2_O_2_ from O_2_. This intrinsic ability to produce H_2_O_2_ is considered a rescue mechanism because it can provide the essential cosubstrate to initiate the catalytic cycle when external H_2_O_2_ is very limited or absent.^171,173^ Even in reactions involving a substrate, LPMOs that do not bind the substrate may still generate H_2_O_2_. This H_2_O_2_ can then fuel the H_2_O_2_-driven reactions catalyzed by substrate-bound LPMOs. A distinctive AA16 family LPMO has been identified that, although inactive toward structural polysaccharides, can produce H_2_O_2_ in the presence of a reductant and O_2_, thus fueling the catalytic activity of AA9 LPMOs.^174^

As early as 2017, Bissaro et al. demonstrated that the addition of horseradish peroxidase (HRP), which consumes H_2_O_2_, inhibited LPMO activity under standard aerobic conditions.^166^ Later studies provided detailed kinetic data on the single-oxygenation event of LPMO-Cu­(I) with O_2_ and H_2_O_2_, revealing that the reoxidation rate of LPMO-Cu­(I) with H_2_O_2_ is at least 2000 times faster than with O_2_.^172^ It has also been reported that in the presence of carbohydrate substrates, H_2_O_2_ accelerated the reoxidation process, while the reoxidation rate of O_2_ slowed down. Moreover, for both bacterial and fungal LPMOs, the oxidation of LPMO–Cu­(I) proceeds several orders of magnitude faster with H_2_O_2_ than with O_2_.^172,175^ Growing experimental evidence has led to a paradigm shift, with many researchers now considering LPMOs as peroxygenases rather than monooxygenases, due to their preferential and highly efficient use of H_2_O_2_.^176,177^

### 4.3 LPMO Electron Donor System

Some LPMOs have been electrochemically characterized, with their reduction potentials typically around +260 mV vs SHE.^152^ The redox potentials may vary depending on the measuring methods used. Recently, Munzone et al. used cyclic voltammetry and redox titration monitored by EPR to determine the Cu­(II)/Cu­(I) redox potential of bacterial *Serratia marcescens* LPMO to be +350 mV vs SHE.^151,178^ Ultimately, LPMO activity is determined by the availability of H_2_O_2_ and the efficiency of its utilization. The latter is governed by the kinetics of the peroxygenase reaction. Seminal studies have established key parameters, such as the rate constant for LPMO reoxidation by H_2_O_2_ and the Michaelis constant for H_2_O_2_, which vary significantly across different LPMOs and are crucial for understanding their in situ catalytic performance.^91,179,180^ Beyond the central role of H_2_O_2_, the presence of diverse electron-donating systems in the lignocellulose degradation network highlights the adaptive versatility of these enzymatic systems (Figure 6). The following section will focus on reductants utilized by LPMO.

**Figure 6:**
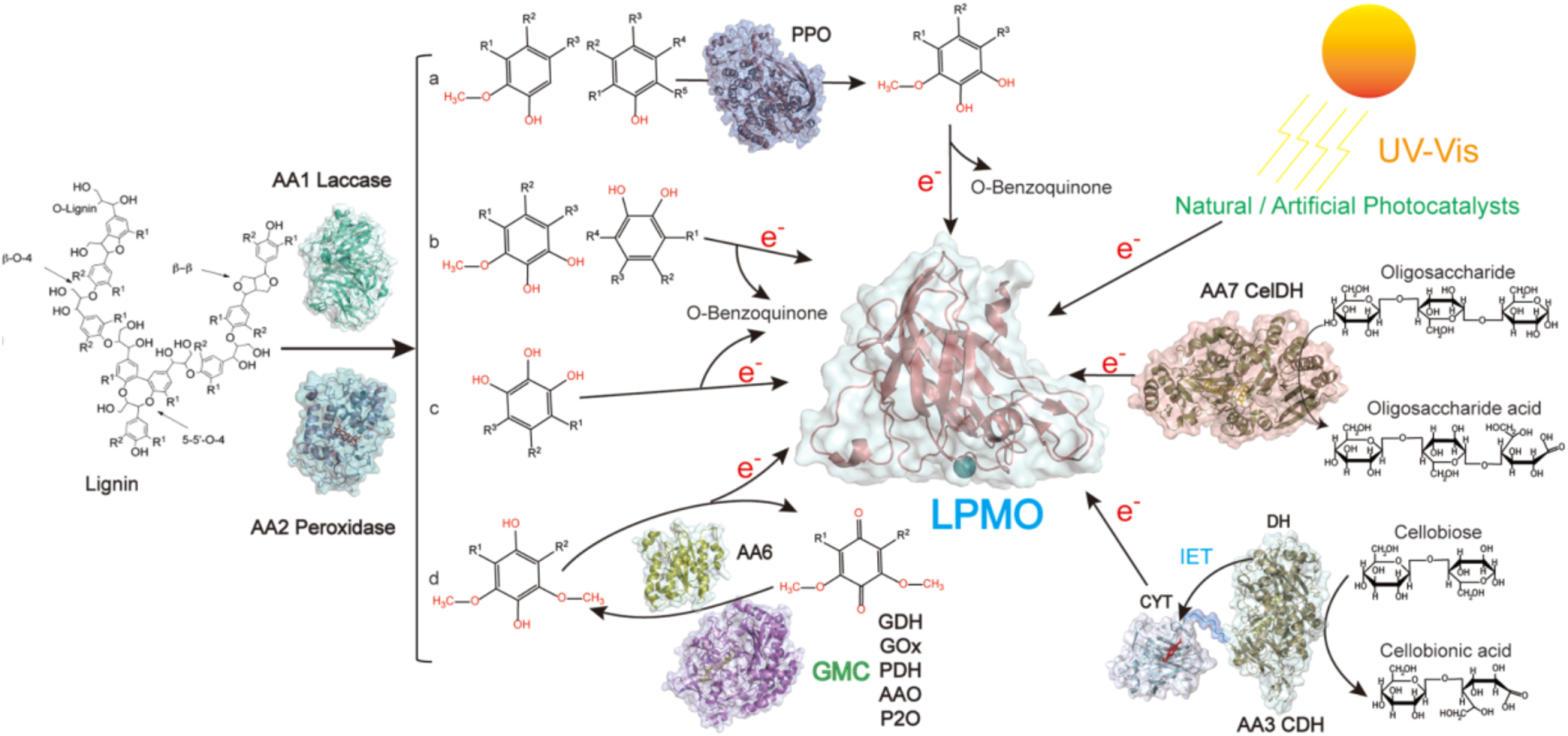
Potential electron donor systems of LPMO in lignocellulose degrading secretoms. a: monophenols, b: pyrocatechol, c: pyrogallol, d: *p*-benzenediol compounds, LPMO: Lytic polysaccharide monooxygenase (PDB: 7OVA), CelDH: Cellooligosaccharide dehydrogenase (PDB: 6YJI), CDH: Cellobiose dehydrogenase (PDB: 4QI6), GMC: Glucose-methanol-choline oxidoreductase (PDB: 4MOE), PPO: Polyphenol oxidase (PDB: 2P3X), Laccase (PDB: 1KYA), Peroxidase (PDB: 1LIP); IET: interdomain electron transfer. As AA11, AA13, AA16, AA17, and AA18 were excluded because their primary activities on chitin, starch, or pectin fall outside the specific scope of lignocellulose degradation covered in this work.

#### 4.3.1 Enzymes that Provide Electrons Directly to LPMO

CDH, a member of the GMC oxidoreductase family, was the first enzyme reported to directly reduce LPMOs and remains the most widely used auxiliary enzyme for *in vitro* studies of LPMO mechanisms.^181,182^ The multicofactor enzyme’s cytochrome domain serves as an electron shuttle to efficiently transfer electron from the catalytic domain of CDH to LPMO (Figure 6).^183,184^ The sequential electron transfer pathway, starting from the substrate cellobiose to the flavin adenine dinucleotide (FAD) cofactor, followed by heme *b*-mediated shuttling to the Cu^2+^ center, constitutes the extracellular electron transport chain. Studies on the genomes and transcriptomes of lignocellulose-degrading microorganisms support the native cooperation between CDHs and LPMOs in nature.^181,185^ The observed coexpression of LPMO and CDH in *Rasamsonia emersonii* on lignocellulosic substrates is consistent with a functional interplay between these enzymes in natural ecosystems.^186^ Furthermore, heterologous expression of *Trichoderma reesei* CDH in a filamentous fungus naturally lacking CDH confirmed in vivo that CDH can serve as an effective electron donor for LPMOs.^187^ These findings underscore the prevalent synergistic interplay between CDHs and LPMOs.

While CDH is a natural partner for LPMOs, its widespread industrial adoption remains limited by operational constraints and high manufacturing costs compared to bulk cellulases.^141^ Current commercial enzyme blends favor more economical reductants or alternative oxidoreductases to maintain H_2_O_2_ levels. The synergistic electron coupling between CDH and LPMO provides a functional basis for designing biomimetic cascades in lignocellulose biorefineries. By leveraging the internal redox balance of this system, industrial processes could potentially minimize the requirement for exogenous reductants while maximizing polysaccharide conversion efficiency. Future efforts leveraging bioengineering strategies such as microbial coexpression^188^ and immobilized multienzyme cascades^189^ are poised to harness this synergy, facilitating more efficient and sustainable biomass conversion.

In addition to CDH, a novel oligosaccharide-oxidizing flavo-enzyme (CelDH; EC 1.1.99.-), which belongs to family AA7, has been recently identified and characterized.^90^ By using X-ray diffraction, kinetic analysis and NMR analysis, it was proven that this FAD-containing CelDH can directly transfer electrons to LPMO without further mediator domains. This discovery is surprising and expands the redox-profiles of oxidative conversion of lignocellulose. Interestingly, as early as 2016, Garajova et al. reported that single-domain flavin enzymes, such as glucose dehydrogenase (GDH) and aromatic alcohol quinone reductase, could serve as limited electron donors for LPMO.^190^ Although the mechanism of electron transfer from FAD to the LPMO copper center remains unresolved, the generation of H_2_O_2_ appears to be the critical step.

#### 4.3.2 Small Molecule Reductants of LPMO

A diverse series of compounds, primarily derived from plant phenolic and lignin’s structural units, such as 1,2-dihydroxybenzene, 1,4-dihydroxybenzene or 1,2,3-trihydroxybenzene serve as natural reductants of LPMOs (Figure 6).^191,192^ Benzoquinone is a well-known electron shuttle *in vivo* and *in vitro*, which is of a certain an efficient electron donor for LPMO.^193,194^ Systematic studies uncovered that other phenolic compounds with structural features such as 1,2-dihydroxy, 1,2-dihydroxy-6-methoxy or 1,2,3-trihydroxy groups can effectively reduce LPMOs.^15,195^ These compounds are primarily derived as intermediates from lignin degradation.^119^ Additionally, some compounds that do not possess the aforementioned features, such as ascorbic acid, glutathione, l-cysteine, and lignin itself, have also been shown to be able to reduce LPMOs.^196,197^

In comparison, monophenolic compounds typically serve as poor electron donors for LPMOs due to their high oxidation potentials. Phenolic compounds containing a second hydroxyl group attached to the benzene ring exhibit relatively low redox potentials (≤250 mV), while monophenolic compounds show higher redox potentials (≥400 mV),^15^ which generally limits their efficiency. Furthermore, some reducing agents, such as gallic acid and ascorbic acid, may not only serve as electron donors under natural conditions but can also generate H_2_O_2_ in the presence of metal ions during *in vitro* experiments, thereby boosting LPMO activity significantly.^179,196^ Reductants affect H_2_O_2_ levels in three ways that vary between reductants: (1) affecting the oxidase activity of the LPMO; (2) abiotic oxidation; (3) abiotic reactions with H_2_O_2_. Additionally, high concentrations of reducing agents, particularly those with lower redox potentials, can deactivate LPMOs within minutes by generating reactive oxygen species (ROS).^166^

#### 4.3.3 Enzymes that Indirectly Provide Electrons to LPMO

Apart from the previously mentioned CDH, other enzymes from the GMC oxidoreductase superfamily have only FAD as the cofactor. These flavoenzymes share the same structural scaffold but with different substrate specificity, including glucose oxidase (GOX; EC 1.1.3.4), GDH (EC 1.1.5.9), pyranose dehydrogenase (PDH; EC 1.1.99.29), and AAO (EC 1.1.3.7). Studies have experimentally proven that GMC oxidoreductases can transfer electrons to LPMO through plant-derived phenolics or lignin structural units, primarily quinones, as electron mediators (Figure 6).^15,198^

Besides GMC oxidoreductases, PPOs (EC 1.10.3.2) have also been demonstrated to activate LPMOs via mediators. More specifically, *Mt*PPO7 from *Myceliophthora thermophila* catalyzes the conversion of guaiacol, a lignin monomer, into a compound containing a 1,2-dihydroxy functional group, which acts as an effective electron donor for LPMO. Thereby enhancing the catalytic activity of LPMO from *Neurospora crassa* for cellulose degradation (Figure 6).^195^ Transcriptomic analyses of the fungi *Irpex lacteus* and *Dichomitus squalens* revealed a strong correlation between the expression of LPMOs and lignin-degrading enzymes, pointing to a putative functional cooperation in the codegradation of lignocellulosic biomass.^137^ Additionally, peroxidases such as MnP (EC 1.11.1.13), LiP (EC 1.11.1.14), VP (1.11.1.16), and laccase are known to oxidize plant phenols and lignin, generating active phenolic compounds that can fuel LPMO. It has been demonstrated that LMS can partially degrade lignin into small molecular weight compounds, which can then act as electron donors for LPMOs.^199,200^

#### 4.3.4 Light-Driven Nonenzymatic Activation of LPMO

In 2016, two pioneering studies established the principle of light-driven LPMO activation: Cannella et al. reported that photosynthetic pigments could serve as effective electron donors for LPMO, and Bissaro et al. demonstrated similar electron transfer using a V-TiO_2_ photocatalys.^201,202^ Another study has shown that melanin produced by *Aspergillus nidulans* under light-induced conditions can also function as an effective electron donor for LPMO, providing experimental evidence for a potential light-driven mechanism that could occur in natural fungal habitats (Figure 6).^203^ A study on LPMO from *Aspergillus fumigatus* explained that light boosts LPMO’s activity through photoexcited chlorophyllin, which transfers electrons to the active site copper ion (Cu^2+^ → Cu^+^), which synergizes with H_2_O_2_ generation by reducing agents.^204^

Beyond electron donation, photocatalytic systems also generate H_2_O_2_ to drive LPMO reactions, and all observed light effects are essentially a consequence of light-promoted H_2_O_2_ formation.^205,206^ However, excessive H_2_O_2_ can lead to self-inactivation of LPMOs. A recent study simulated natural conditions, specifically a lignocellulosic mixture, showing that in the presence of lignin, light can activate LPMOs.^207^ Lignin irradiation promotes H_2_O_2_ production, which drives the redox reactions of LPMOs. However, high concentrations of H_2_O_2_ inhibited saccharification efficiency.^206^ In very few natural habitats light-driven systems could constitute a primary auxiliary system for LPMO, but it could be designed and optimized for biotechnological applications.

### 4.4 Novel LPMO-Like Proteins

Reflecting the broadening landscape of prokaryotic oxidative enzymes (as noted in Section 2), a newly reported lytic cellulose monooxygenase (EC 1.14.99.54), CelOCE, from the metagenome of lignocellulose-degrading microbial communities exhibits unprecedented mechanisms for cellulose oxidation.^142^ Detailed structural and biochemical analyses reveal that CelOCE operates via an exotype mechanism exhibiting strict C1 regioselectivity, which contrasts with the endopreference of many LPMOs. This unique mode of action results in exclusive production of cellobionic acid rather than a mixture of oxidized oligosaccharides. The crystal structure indicates a homodimeric configuration with a buried copper active site within a compact jellyroll scaffold, further distinguishing it from the typical LPMO fold. Notably, despite these structural differences, CelOCE shares the fundamental catalytic logic with LPMOs: it is also a copper-dependent peroxygenase whose oxidative activity requires both prior reduction of the active-site copper and the presence of H_2_O_2_ as a cosubstrate.^142^ The secretome of an engineered *Trichoderma reesei* strain expressing this metalloenzyme boosted the glucose release by 21% under industrially relevant conditions, concretely demonstrating its significant biotechnological potential for cellulose valorization..

Genomic and transcriptomic studies uncovered a new family of fungal copper-binding proteins, which share key structural features with LPMOs but lack any polysaccharide oxidative activity.^208^ These proteins are widely distributed in the fungal kingdom and are also found in yeasts that do not degrade plant biomass. Besides the conserved “histidine brace”, the LPMO-like proteins contain an additional Asp ligand to Cu­(II). Structural analysis showed that these LPMO-like proteins lack carbohydrate-binding modules but have a GPI anchor, indicating membrane association. In *Laccaria bicolor*, the LPMO-like protein is upregulated during symbiosis with tree roots, suggesting a role in fungal morphogenesis or cell wall remodeling, rather than biomass degradation. These findings serve as an important reminder that the presence of a conserved “histidine brace” copper site is not a definitive predictor of polysaccharide oxidative activity.^209^ Collectively, these results reveal the presence of LPMO-like copper-binding proteins in nature that do not function in oxidative carbohydrate degradation.

## 5 Nonenzymatic Polymer Degradation

The Fenton reaction, which involves the reaction of H_2_O_2_ with reduced transition metals is another strategy employed by microorganisms for lignocellulose degradation.^210,211^ Enzymes typically range in size from 20 to 100 Å, whereas the pore size of lignocellulose is generally between 10 and 40 Å, thereby restricting enzyme penetration and limiting effective access to the plant cell wall matrix.^91^ *In situ* generated hydroxyl radicals can nonspecifically oxidize the structure polysaccharides and lignin, which play a key role in the initial nonenzymatic pretreatment of lignocellulose.^212^ However, hydroxyl radicals are highly reactive and destructive and can cause irreversible damage to microbial cells, posing a high risk for the microorganism.^213^ Therefore, the Fenton reaction must be activated at a certain distance apart from the fungal hyphae to prevent damage. To achieve this spatial control, fungi produce hydroxyl radicals in a controlled and spatially targeted manner, resulting in measurable differences in the Small Angle X-ray Scattering and Wide-Angle X-ray Scattering spectra.^214^ Fungi regulate the Fenton reaction by secreting metal ion chelators, such as oxalates. At low concentrations, oxalic acid promotes the Fenton reaction, whereas at high concentrations it suppresses it, due to pH gradient formation and the shift from iron-mobilizing Fe^3+^–oxalate to Fenton-inhibiting Fe^3+^/Fe^2+^–(oxalate)_2_,_3_ complexes.^215^ In addition, by secreting organic acids, fungi lower the local pH to 3.5–5, optimizing Fe^3+^ solubility for the Fenton reaction and simultaneously maintaining enzyme stability.^216^

Brown-rot fungi have been most deeply studied for using the Fenton reaction in lignocellulose degradation. Increasing evidence indicates that brown-rot fungi mediate the Fenton reaction through two main mechanisms: enzymatic pathways involving oxidoreductases and low-molecular-weight redox-active metabolite mediated pathways.^63,217^ The enzymatic pathway relies on oxidoreductases capable of reducing O_2_ and Fe^3+^. This group includes oxidoreductases from the AA3, AA4, and AA5 families, which produce H_2_O_2_, as well as iron-reducing enzymes from the AA8 family.^218^ The low-molecular-weight redox-active metabolite mediated pathway involves electron shuttling from central cellular metabolism to secreted low-molecular-weight reductants. These reductants subsequently transfer electrons to O_2_ or Fe^3+^ in close proximity to lignocellulosic chains, triggering localized Fenton reactions. Among these low-molecular-weight reductants, dimethoxyhydroquinone has been extensively studied, which forms a redox cycle mediated by AA6-benzoquinone reductase (Figure 6).^219^ Interestingly, the product para-hydroxyphenolic compounds are then integral to the electron donor systems supporting LPMO activity. Another study employed an LPMO/GDH system that harnesses quinone redox cycling to enhance Fe^3+^ reduction and H_2_O_2_ production, thereby fueling the Fenton reaction.^220^ The concentration of H_2_O_2_ directly influences the rate of the Fenton reaction, making it a critical factor to consider. However, *in situ* analyses of the Fenton reaction mechanisms in fungi still present a significant challenge.^221^

## 6 H_2_O_2_ as a Dynamic Regulator

In the previous chapters, we discussed two distinct oxidative enzyme systems and one nonenzymatic system involved in lignocellulose degradation. H_2_O_2_ interlinks three oxidative systems as the central molecule, forming a complex network. In the subsequent section, the spatiotemporal dynamic regulation of H_2_O_2_ will be discussed in detail to provide perspectives on the lignocellulose degradation in natural environments.

The following section details the dedicated enzymatic systems primarily responsible for H_2_O_2_ generation, excluding LPMOs. Within the oxidative secretome, the primary physiological function of LPMOs is to act as H_2_O_2_-dependent peroxygenases that facilitate glycosidic bond cleavage. Their capacity to generate H_2_O_2_ via an oxidase pathway, a phenomenon predominantly observed during incubation with lignin-derived reductants in substrate-depleted environments,^62,173^ represents a secondary, condition-dependent reaction.

### 6.1 Enzymes Producing H_2_O_2_

As previously outlined within the secretome of lignocellulolytic species, several extracellular enzymes can produce H_2_O_2_, most of which belong to the GMC oxidoreductase superfamily. These enzymes can be divided into two categories based on their substrates: lignin-driven and polysaccharide-driven. The polysaccharide-driven enzymes include CDH, GOX, pyranose oxidase (P2O), and glucooligosaccharide oxidase (GOOX). The lignin-driven enzymes include AAO, MOX, and vanillyl alcohol oxidase (VAO). Additionally, glyoxal oxidase (GLOX) catalyzes the oxidation of low molecular weight aldehydes, such as glyoxal and methylglyoxal, which are byproducts of fungal metabolic pathways (Figure 7).

**Figure 7:**
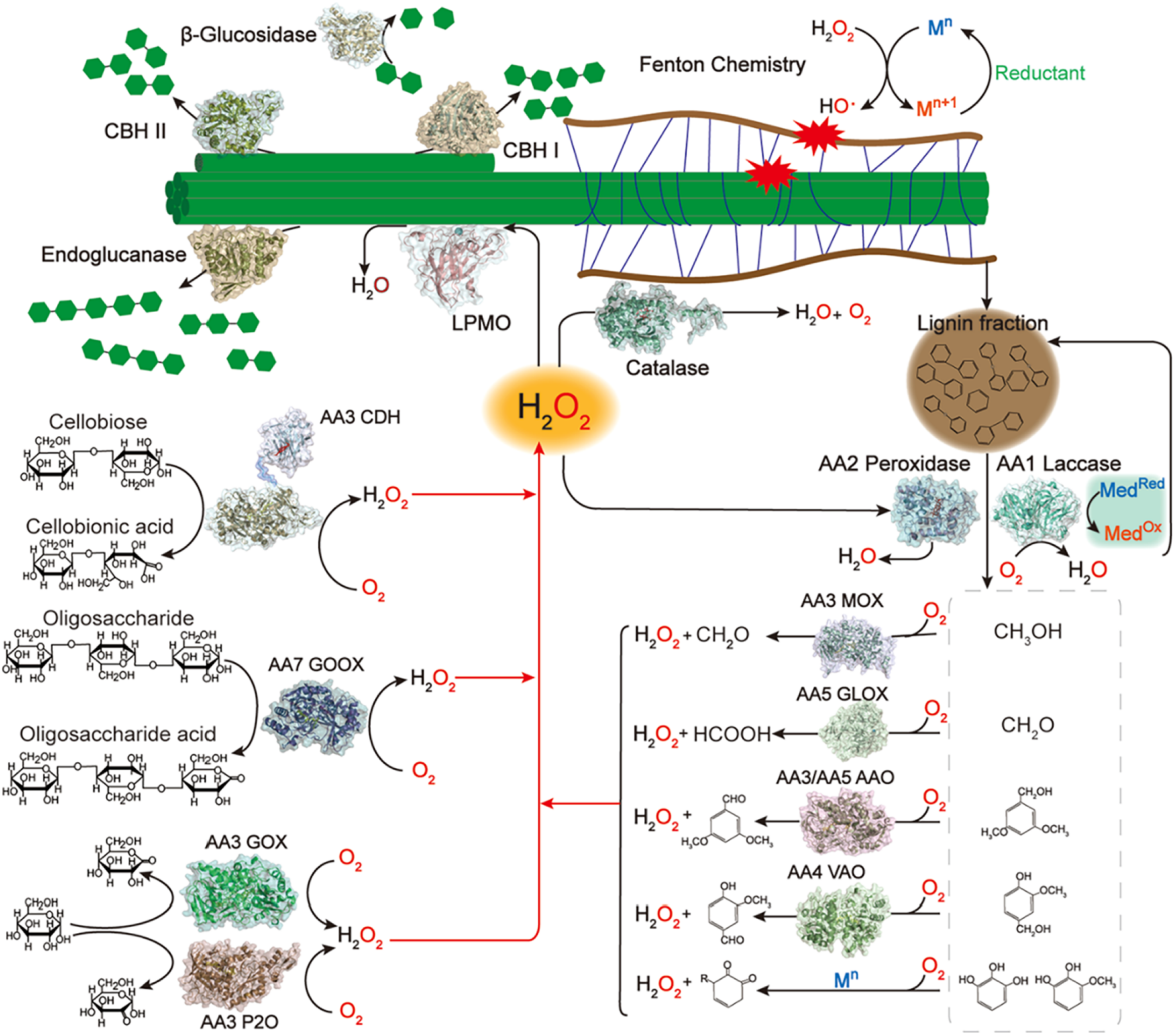
Schematic diagram of lignocellulose oxidative degradation network. Adapted and expanded from Bissaro et al.^91^ Med: Redox Mediator; M^n^: Metal ion; CBH: Cellobiohydrolase (PDB: 4AVN); GOOX: Glucooligosaccharide oxidase (PDB: 1ZR6); GLOX: Glyoxal oxidase (PDB: 5C92); VAO: Vanillyl-alcohol oxidase (PDB: 1AHU); MOX: Methanol oxidase (PDB: 6H3G); AAO: Aryl alcohol oxidase (PDB: 3FIM); CDH: cellobiose dehydrogenase (PDB: 4QI6); GOX: Glucose oxidase (PDB: 1CF3); P2O: Pyranose oxidase (PDB: 1TZL); GDH: Glucose dehydrogenase (PDB: 4YNT); LPMO: Lytic polysaccharide monooxygenase (PDB: 6YDC); Laccase (PDB: 1KYA), Peroxidase (PDB: 1LIP).

These H_2_O_2_-producing enzymes are widely present in both white-rot and brown-rot fungi, highlighting that H_2_O_2_ is produced regardless of the degradation strategy used by the fungi. For the lignocellulose conversion of white-rot fungi, the major importance of peroxidases and H_2_O_2_-producing machinery was confirmed by temporal transcriptome analysis of *Obba rivulosa*.^222^ An alcohol oxidase (AOX) belonging to GMC oxidoreductases was highly expressed, which was the most abundant transcript after two AA2MnPs among lignin degradation-related transcripts. Another proteome and transcriptome study on white-rot fungus *Phlebia radiata* supported that the H_2_O_2_-producing enzymes, such as GLOX and AOX, are highly coordinated in time with H_2_O_2_-dependent enzymes like AA2-family peroxidases and LPMOs.^223^ Different to lignin-degrading white rot ancestors, brown rot fungi often decompose wood faster, by selectively removing carbohydrates and leaving most lignin behind. Directed culture and spatial transcriptome analysis of the brown-rotten fungus *Postia placenta* revealed an oxidative-hydrolytic two-step mechanism: At the mycelial front (0–5 mm), a distinct oxidative phase is initiated through the synchronized upregulation of 549 genes, primarily encoding H_2_O_2_-generating oxidases and LPMOs. This is followed by a spatially decoupled hydrolytic phase in the distal regions (15–35 mm), characterized by the induction of 659 CAZyme genes, including various glycoside hydrolases (GHs).^29^ This mechanism reflects a strategic segregation of oxidative and hydrolytic events that optimizes the efficiency of lignocellulose deconstruction by brown-rot fungi.

H_2_O_2_-producing enzymes act on different compounds derived from different stages of lignocellulose degradation. MOX, for example, oxidizes methanol produced during lignin demethylation, providing a major source of H_2_O_2_ for the Fenton reaction in brown rot fungi.^224^ AAO, one of the most prevalent GMC oxidoreductases in lignin-degrading microbes, acts on lignin degradation intermediates such as 3,5-dimethoxybenzyl alcohol, producing H_2_O_2_ simultaneously.^225^ GOX and P2O act on the same substrate, pyranose from cellulose degradation, but oxidize at different positions of the sugar.^226^ Both enzymes can reduce quinones, which are redox-active compounds served as key mediators in the degradation of lignocellulose and a product of lignin degradation.

More studies have shown that these enzymes demonstrate a synergistic interplay, characterized by the complementary nature of their functions and the cohesive coordination of their catalytic mechanisms. Aldehydes released by AOX are the substrates for GLOX, which is found widely in wood-degrading fungi.^227^ VAO, a member of the 4-phenol oxidase family (AA4), is mainly found in lignin-degrading ascomycetes. It can oxidize lignin depolymerization products like guaiacol and has also additional activities, including dehydrogenation, hydroxylation, deamination, and benzyl ether cleavage.^228^ The flavoenzyme GOOX has the same catalytic mechanism as CDH, P2O, and GOX, but with different substrate specificities.^229,230^ They primarily act on glucose oligosaccharides and some of them show activity on xylose oligosaccharides as well.^231^ Given the similarity in substrates of GOOX and LPMO, in natural habitats, GOOX may be situated closer to LPMO, directly serving as the oligosaccharide chain produced by LPMO cleavage, and generating H_2_O_2_ to further drive the reaction of LPMO.^91^ Besides and as already described in the previous section, a newly discovered CelDH, which belongs to the same family as GOOX, has been proved to be able to transfer electron directly to LPMOs.^90^

### 6.2 Utilization and Elimination of H_2_O_2_

Besides the enzymatic consumption by LPMO or peroxidases, H_2_O_2_ can also react with reductive metal ions, leading to the generation of hydroxyl radicals via the Fenton reaction and initiate nonenzymatic lignocellulose degradation.^90^ Chang et al. constructed an *in vitro* lignocellulose cell wall degradation system. Cellulases, GMC oxidoreductases, and LPMOs were used to mimic fungal extracellular degradation. They monitored H_2_O_2_ concentration on the plant cell wall surface with an ultramicroelectrode.^169^ The results showed that LPMO scavenges generated H_2_O_2_, indicating a local H_2_O_2_ homeostasis where enzymatically produced H_2_O_2_ is quickly used by another enzyme in this microenvironment. These H_2_O_2_ utilizers together with the H_2_O_2_ producers forms a coherent and robust enzymatic system for oxidative degradation of lignocellulose (Figure 7).

Additionally, microorganisms have also evolved a critical adaptive mechanism to circumvent challenges associated with H_2_O_2_, using catalase to disproportionate H_2_O_2_ into O_2_ and H_2_O. It has been found in nearly all forms of life.^232^ The low affinity of catalases for H_2_O_2_ (compared to other H_2_O_2_-dependent enzymes) does not inhibit peroxidases and is suited to maintain a H_2_O_2_ concentration within a certain, low threshold. The high catalytic efficiency of catalase for H_2_O_2_ quickly reduces too high H_2_O_2_ concentrations.^91^ Studies have demonstrated that catalase and peroxidases were coexpressed in *P. chrysosporium* during spruce sawdust degradation.^233^ Another phenomenon was observed in *P. placenta* that at the later stages of wood degradation, catalase was coexpressed with GH enzymes to protect GHs from oxidative damage.^29^

## 7 Conclusions and Prospects

This review highlights the action and interaction of oxidoreductases involved in the oxidative enzymatic degradation of lignocellulose from a mechanistic and an evolutionary perspective. The coevolutionary interplay between microorganisms and plants has shaped diverse lignocellulose-degrading systems, with filamentous fungi (white-rot and brown-rot fungi) emerging as primary lignocellulose decomposers within terrestrial ecosystems through a specialized oxidative enzyme arsenal. Key enzymes including laccases, lignin-active peroxidases, and LPMO) form the core of the oxidative degradation networks, synergistically breaking down recalcitrant lignin and polysaccharides. Nonenzymatic degradation, particularly by Fenton reaction generated reactive oxygen species (ROS) and radicals, precede enzymatic depolymerization by initiating structural disruption. H_2_O_2_ acts as a central molecule, interconnecting enzymatic H_2_O_2_ producers (e.g., GMC-oxidoreductases), utilizers (peroxidases, LPMOs), and eliminators (catalases) to maintain spatiotemporal regulation. The integration of enzymatic and nonenzymatic mechanisms, coupled by key molecules like H_2_O_2_, enables efficient depolymerization of lignocellulose and transformation of aromatic compounds. The investigation of these enzymes and reactions is crucial to understand natural biomass depolymerization and to optimize industrial biorefinery processes.

Despite significant advances in recent years, several critical questions remain to be addressed. Natural fungal degradation is slow and. fungi live on their substrates for months and years. Their fine-tuned enzyme secretion orchestrates oxidative and hydrolytic processes to utilize carbon most efficiently while conserving their host structure and defending their niche against competitors. This evolutionary strategy is completely different from our current expectations on biorefinery processes, which require a fast, and efficient conversion resulting in uniform products. Looking forward, we should emulate nature’s choreography, not its objectives. Enzyme engineering optimizes enzyme performance like substrate specificity, thermostability and turnover stability, solvent/ionic-liquid tolerance or product inhibition to improve process economics, which will be benefited greatly from AI-driven or machine learning approaches. The second important optimization lies in the formulation in enzyme cocktails or programmable dosing, which requires further exploration and development. The explicit differentiation of “what fungi aim to achieve with their secretome” from “what we want to obtain using enzymes”, can initiate new approaches to develop biorefinery processes that valorize both carbohydrates and lignin by a fast generation of fractionated product streams that can be converted into high-value materials and molecules.

## Supplementary Material





## Notes

Multiple sequence alignment files used for phylogenetic tree and sequence logo construction: AA1.fasta, AA2.fasta, AA9.fasta, AA10.fasta, AA14.fasta, AA15.fasta (FASTA) (ZIP)
Bioinformatics analysis workflow (Figure S1); extended sequence logo of long-range electron transfer chain and cofactor coordination of lignin active peroxidases (Figure S2) (PDF)
