# Supplementary material for: Plant Biomass Depolymerization by Oxidoreductases: Structural Characteristics, Synergy Mechanism, and Evolutionary Adaptation
Source: J Agric Food Chem. 2026 Jun 25;74(26):20104–25. doi: 10.1021/acs.jafc.5c16214 (PMC13353379; doi:10.1021/acs.jafc.5c16214)
Supplement: Supplementary file 2 [file jf5c16214_si_002.pdf]

Supported Information

Plant biomass depolymerization by oxidoreductases: structural characteristics, synergy mechanism and evolutionary adaptation

Xiaoyu Ma[1], Florian Csarman[2], Yunjia Guan[1], Lushan Wang[1], Roland Ludwig[2*], Su Ma[1*]

[1] State Key Laboratory of Microbial Technology, Shandong University, Binhai Road 72/N2, 266237 Qingdao, China

[2] Institute of Food Technology, Department of Biotechnology and Food Science, BOKU University, Muthgasse 11, 1190 Vienna, Austria

**Email: roland.ludwig@boku.ac.at; masu@sdu.edu.cn*

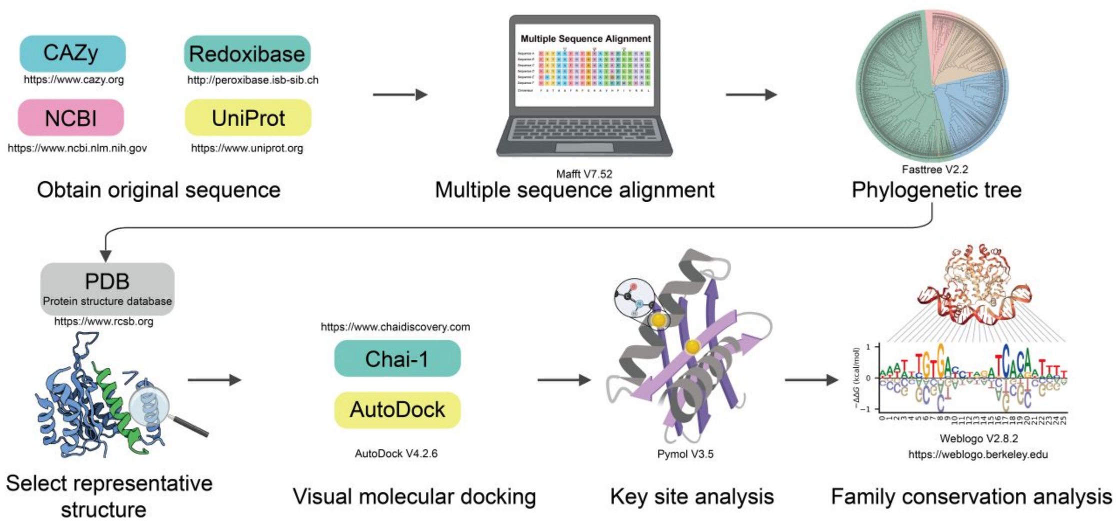

Figure S1. A detailed illustration of the bioinformatics analysis workflow. Original enzyme sequences were retrieved from multiple specialized databases (e.g., RedoxiBase,[1] CAZy database[2]). Multiple sequence alignments were performed using MAFFT,[3] and phylogenetic trees were constructed with FastTree.[4] Representative sequences with available crystal structures were then selected from the phylogenetic clusters. Protein-substrate complex structures were obtained through molecular docking (e.g., using Chai-1[5]) with selected substrate molecules. Key amino acid residues at the active site and substrate-binding interface were identified and visualized using PyMOL software. Finally, sequence logos were generated using WebLogo to analyze the conservation patterns of critical residues across each enzyme family.[6]

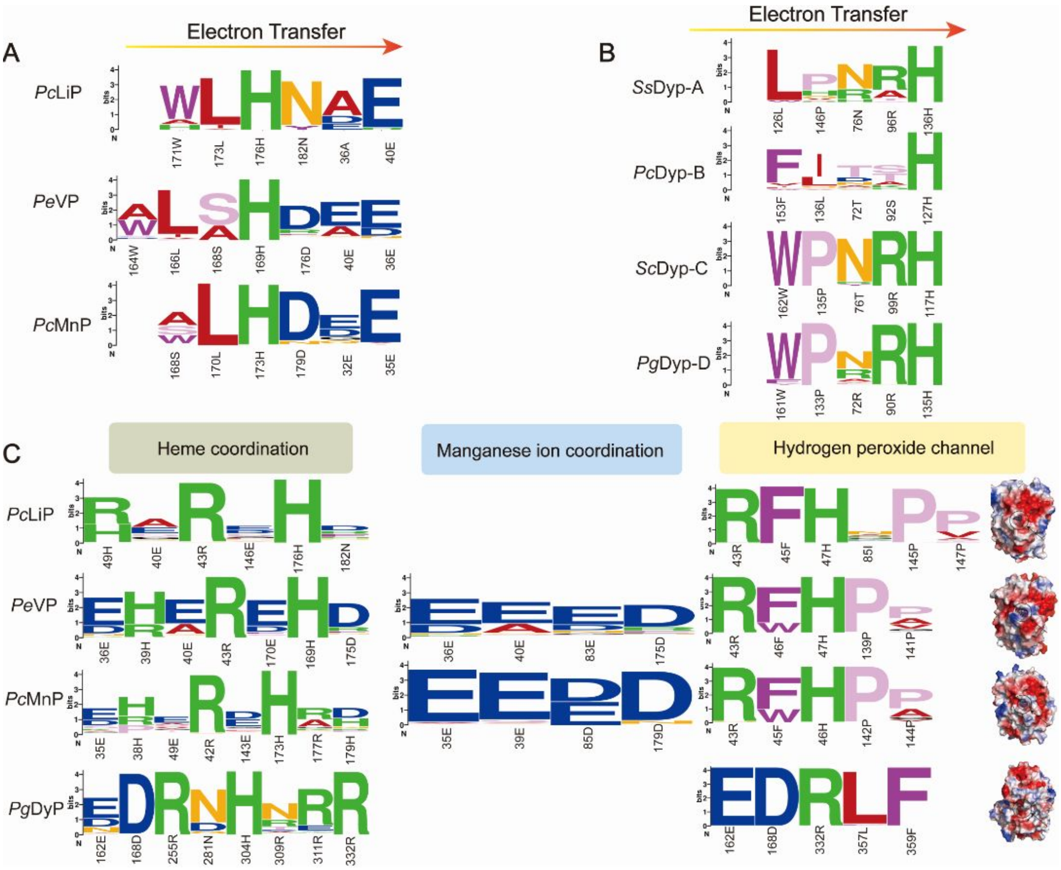

Figure S2. **Extended sequence logo of key residues in the long-range electron transfer chain and cofactor coordination of lignin active peroxidases.** (A, B) Extended sequence logo showing all amino acid residues constituting the long-range electron transfer pathways in different lignin-active peroxidases.[7] These panels provide an expanded view of the conserved residues highlighted in Figure 4. (C) Sequence logo of amino acid residues involved in cofactor (heme) coordination. LiP: lignin peroxidase (1LIP); MnP: manganese peroxidase (PDB: 1YYD); VP: versatile peroxidase (PDB: 2BOQ); DyP: dye decolorization peroxidase (4W7J). Sequences used for sequence logo generation are provided in a separate FASTA file.

Reference

1. Savelli, B.;Li, Q.;Webber, M.;Jemmat, A. M.;Robitaille, A.;Zamocky, M.;Mathé, C.,Dunand, C., Redoxibase: A database for ros homeostasis regulated proteins. *Redox Biol* **2019,** *26*, 101247.
2. Lombard, V.;Henrissat, B.,Garron, M.-L., Cazac: An activity descriptor for carbohydrate-active enzymes. *Nucleic Acids Res.* **2024**, gkae1045.
3. Rozewicki, J.;Li, S.;Amada, K. M.;Standley, D. M.,Katoh, K., Mafft-dash: Integrated protein sequence and structural alignment. *Nucleic Acids Res.* **2019,** *47* (W1), W5-W10.
4. Price, M. N.;Dehal, P. S.,Arkin, A. P., Fasttree: Computing large minimum evolution trees with profiles instead of a distance matrix. *Mol Biol Evol* **2009,** *26* (7), 1641-50.
5. Discovery, C.;Boitreaud, J.;Dent, J.;McPartlon, M.;Meier, J.;Reis, V.;Rogozhnikov, A.,Wu, K., Chai-1: Decoding the molecular interactions of life. *bioRxiv* **2024**, 2024.10.10.615955.
6. Crooks, G. E.;Hon, G.;Chandonia, J. M.,Brenner, S. E., Weblogo: A sequence logo generator. *Genome Res* **2004,** *14* (6), 1188-90.
7. Ruiz-Dueñas, F. J.;Morales, M.;García, E.;Miki, Y.;Martínez, M. J.,Martínez, A. T., Substrate oxidation sites in versatile peroxidase and other basidiomycete peroxidases. *J. Exp. Bot.* **2009,** *60* (2), 441-452.
